# A Numerical Investigation of a Melting Rate Enhancement inside a Thermal Energy Storage System of Finned Heat Pipe with Nano-Enhanced Phase Change Material

**DOI:** 10.3390/nano12152519

**Published:** 2022-07-22

**Authors:** Anuwat Jirawattanapanit, Aissa Abderrahmane, Abe Mourad, Kamel Guedri, Obai Younis, Belgacem Bouallegue, Khanyaluck Subkrajang, Grienggrai Rajchakit, Nehad Ali Shah

**Affiliations:** 1 Department of Mathematics, Faculty of Science, Phuket Rajabhat University (PKRU), Phuket 83000, Thailand; anuwat.j@pkru.ac.th; 2 LPQ3M, Université Mustapha Stambouli de Mascara, Mascara 29000, Algeria; a.aissa@univ-mascara.dz (A.A.); mourad.abed@univ-mascara.dz (A.M.); 3 Mechanical Engineering Department, College of Engineering and Islamic Architecture, Umm Al-Qura University, Makkah 21955, Saudi Arabia; kmguedri@uqu.edu.sa; 4 Department of Mechanical Engineering, College of Engineering at Wadi Addwaser, Prince Sattam Bin Abdulaziz University, Al-Kharj 11991, Saudi Arabia; oubeytaha@hotmail.com; 5 College of Computer Science, King Khalid University, Abha 61413, Saudi Arabia; bbelgacem@kku.edu.sa; 6 Faculty of Science and Technology, Rajamangala University of Technology Suvarnabhumi, Nonthaburi 11000, Thailand; 7 Department of Mathematics, Faculty of Science, Maejo University, Chiangmai 50100, Thailand; kreangkri@mju.ac.th; 8 Department of Mechanical Engineering, Sejong University, Seoul 05006, Korea; nehadali199@sejong.ac.kr

**Keywords:** shell-and-tube TES, nano-enhanced PCM, nanoparticles, fins, latent heat thermal energy storage (LHTES)

## Abstract

Thermal energy storage via the use of latent heat and phase transition materials is a popular technology in energy storage systems. It is vital to research different thermal enhancement techniques to further improve phase transition materials’ weak thermal conductivity in these systems. This work addresses the creation of a basic shell and a tube thermal storage device with wavy outer walls. Then, two key methods for thermal augmentation are discussed: fins and the use of a nano-enhanced phase change material (NePCM). Using the enthalpy–porosity methodology, a numerical model is developed to highlight the viability of designing such a model utilizing reduced assumptions, both for engineering considerations and real-time predictive control methods. Different concentrations of copper nanoparticles (0, 2, and 4 vol%) and wavenumbers (4,6 and 8) are investigated in order to obtain the best heat transmission and acceleration of the melting process. The time required to reach total melting in the studied TES system is reduced by 14% and 31% in the examined TES system, respectively, when NePCM (4 vol% nanoparticles) and N = 8 are used instead of pure PCM and N = 4. The finding from this investigation could be used to design a shell-and-tube base thermal energy storage unit.

## 1. Introduction

Energy consumption continues to rise year after year as a result of rising worldwide demand, as shown by data released by International Energy Agencies [1,2]. Increased energy use from conventional energy sources such as fossil fuels, on the other hand, is both unsustainable and very detrimental to the environment. To address rising energy demand, researchers have focused on the need to develop clean and renewable energy sources and associated technologies [3,4,5]. In recent years, solutions such as the use of better fluids and energy storage devices have been investigated [6,7,8,9]. Thermal energy storage (TES) is a critical subsystem in solar energy applications. It enables the power plant to be self-sufficient during periods of low or no solar radiation intensity, allowing power generation to continue for an extended period of time and thus increasing system reliability [10,11,12]. Three forms of TES exist in practice: sensitive, latent, and thermochemical TES. The latent TES using phase change materials (PCMs) is believed to be more favorable than the others because of PCMs’ high energy storage-to-volume ratio and their absence of or minor temperature changes during operation. Consequently, PCM-based TES systems are applicable to a broad scope of use, such as energy conservation in buildings, home hot water storage tanks and air conditioning units, waste heat recovery, and thermal management applications [13,14,15,16]. Ge et al. [17] presented an autonomous temperature adjustment technique based on the absorption of transitory heat by low melting point PCM. Their testing revealed that 3.4125 mL of PCM was sufficient to keep the module below 45 °C for 16 min at a power output of 2.832 W. Arshad et al. [18] explored the heat transmission performance of a heat sink equipped with fins and loaded with PCM (n-eicosane) in order to establish its applicability for passive cooling of electronic devices by examining its heat transmission performance. The findings given in their investigation indicated that the low-temperature PCM used in their study maintained the heat sink base temperature within acceptable ranges. Fu et al. [19] examined the effectiveness of a multilayer-structured photovoltaic thermal (PVT) mechanism using PCMs. Their findings indicated that by including a heat exchanger and a photovoltaic/thermal mechanism, the mean PV performance of the photovoltaic/thermal mechanism might be increased by around 1%. Due to gallium’s (PCM) better thermal transport qualities, Peng et al. [20] suggested gallium for spacecraft thermal energy storage in microgravity. They discovered that when gallium was used in microgravity instead of conventional PCM such as ice and n-octadecane, it reduced the time required by the melting process by 88.3% and 96.4%, respectively, while boosting the capacity of total energy storage by 20.7% and 123.3%. Mahani et al. [21] investigated the transient behavior of the melting process within a Latent Heat Storage system using zigzag plates. They discovered that the zigzag structure achieves more improvement than the smooth form. To establish guidelines for the safe and reliable usage of latent-type TES, Juyoung Oh et al. [22] investigated the compatibility of several TES material compositions with a heat transfer fluid (HTF). Memme et al. [22] examined optimizing the size of TES using real-world data analysis and establishing economic and energy parameters that may result in cost savings and reduced greenhouse gas emissions.

Due to the fact that the thermophysical properties of PCMs fluctuate after numerous working cycles and their very low thermal conductivity, the rate of energy storage and release is reduced, limiting their widespread use and commercialization [23,24]. To overcome PCM’s restrictions, heat transmission between the PCM and its container must be increased. Heat conductivity is enhanced by loading the PCM with porous materials or nanoparticles. Additionally, increasing the heat transfer area by encapsulation, metal foam, or fins may significantly improve TES performance [25,26,27]. Kok et al. [28] developed a new design for heat transmission fins to aid in the melting process. The findings showed that by including the proposed fin in the heat storage tank, the melting time was reduced by 63%. Sun et al. [29] suggested and assessed novel fin architectures for PCM-based battery thermal control systems. They observed that the PCM-Fin system is capable of regulating the battery’s temperature increase even at a heat production rate of 20 W. Shehzad et al. [30] investigated the impact of fin inclination and placement on the free convection of aqueous-based nano-encapsulated PCMs in a fin-equipped heat exchanger. The findings indicate that vertically oriented fin designs have superior thermal performance. However, the obstruction of flow produced by adjacent fins degrades thermal performance. Sodhi et al. [31] investigated the increase in compound charging and discharging in multi-PCM systems with non-uniform fin arrangement. According to their findings, by combining non-uniform fin allocation and PCM block length ratio optimization for the multi-PCM system, they were able to achieve a 30% and 9% decrement in charging and discharging times, respectively, when compared to the single PCM system. Al-Omari et al. [32] examined the thermal management features of a heat sink loaded with a PCM and proposed a novel fin concept in which the fins are disconnected from the heated sink base. The results indicated that optimizing fin lifting may result in a significant decrease in the values of maximum temperatures and an increment in heat discharge rate. The results indicated that optimizing fin lifting may result in a significant decrease in peak temperatures and an increment in heat discharge rate from the sink. Sarani et al. [33] developed the use of a discontinuous fin arrangement to accelerate solidification in PCM with and without nanoparticles. The outputs demonstrated that converting continuous fins to discontinuous fins improved heat transmission in PCM and that distributing the strips fins optimally saved 89% of the time. Usman et al. [34] investigated the thermal performance of unfinned and finned PCM-based heat sinks with a variety of fin shapes and PCM. According to their findings, RT-44 is shown to be the most successful passive temperature control material for electronic devices, while triangular inline pin fin heat sinks are found to be the most effective heat sink shape. Aly et al. [35] also evaluated the phase transition of a PCM by integrating corrugated fins in a double-tube heat exchanger. According to the findings, the fins lowered solidification time by 30–35% when compared to normal flat fins. Mosavi et al. [36] researched the thermal behavior of a PCM-based heat sink with horizontal fins. Horizontal fins were found to improve thermal conductivity in the system, although this came at the cost of a decrease in latent heat. Five horizontal fins were determined to be the ideal number for fins based on the results.

Moreover, adding highly thermal conductive additives such as nanoparticles into PCMs to generate composites called NePCMs has shown to be a successful technique for increasing PCM thermal conductivity. However, multiple studies have demonstrated that nano-additives increased the viscosity of NePCM, negating some of the benefits of higher thermal conductivity [37,38,39]. Kothari et al. [40] conducted an experimental investigation to determine the influence of nanoparticle volume fraction and fin count on PCM-based heat sinks for temperature control systems. Their findings showed that the highest decrease in melting time was 26%, 13%, and 9% for three-finned, one-finned, and unfinned NePCM-based heat sinks, respectively. Nizetic et al. [41] examined the influence of nanoparticle incorporation on the thermal characteristics of PCM and the usage of NePCM in photovoltaic module cooling, waste heat recovery, and solar collector systems. Babazadeh et al. [42] utilized numerical modeling to determine the rate of heat dissipation from HS with fins in conjunction with NePCM. The nanoparticle size has a considerable influence on the heat transfer rate, and the optimal diameter was determined to be 40 nm when fouling in the heat release unit was considered. Arshad et al. [43] described the synthesis of a number of mono and hybrid NePCMs. Different nanoparticles, such as graphene nanoplatelets (GNPs), multiwall carbon nanotubes (MWCNTs), aluminum oxide (Al_2_O_3_), and copper oxide (CuO), are dispersed in PCM to create hybrid NePCMs. The NePCM composed of 75% GNPs and 25% MWCNTs exhibits the greatest thermal conductivity increase. Sivashankar et al. [44] studied CPV cells employing graphene nanoplatelets integrated into PCM. They reported that the efficiency and output power of the CPV cells improved when NePCM is added to the system, and the optimal volumetric fraction of nanoparticles was determined to be 0.5%. Faraji et al. [45] used numerical analysis to explore the NePCM melting in an inclined rectangular cage. They demonstrated that the particle concentration in PCM has an impact on heat transmission performance. Mhiri et al. [46] developed a unique kind of stable PCM for TES systems comprised of nanocomposites containing a paraffin/graphite combination embedded in carbon foam. The findings indicated that adding graphite and carbon foam to paraffin wax improved its thermal qualities and prevented melted paraffin from escaping, hence preserving its steady thermal performance. Shirazi et al. [47] examined the viability of employing PCM nanocomposites to regulate the heat generated by a Li-ion battery package effectively. According to simulation findings, encapsulating the batteries inside a paraffin nanocomposite reduced temperature variance and increased thermal conductivity. Bondareva et al. [48] explored the thermal properties of a finned heat sink loaded with NePCM. They discovered that the addition of the nanopowders improved the first melting process due to heat conduction between the liquid PCM and the solid. Ghalambaz et al. [49] used the Taguchi optimization technique to optimize the melting process of the NePCM in a shell/tube TES configuration. The findings indicated a 23.3% increase in stored thermal energy (Cu) and a 22.5% increase in stored thermal energy (GO) were attained.

According to prior literature, many investigations have concentrated on improving the melting performance of PCM in a shell-and-tube TES system using simple fins and nanoparticles. However, few studies have focused on employing branching fins and modified shell geometry to improve NEPCM melting proves in the TES unit. Consequently, the primary goal of this paper is to examine how PCM augmented with nanoparticles and branching fins melts in a wavy shell-and-tube thermal energy storage system. As a result, a two-dimensional model was created, which contained a NePCM-loaded finned shell-and-tube LHTES heat exchanger. The melting mechanism of the NePCM in the model is numerically investigated, focusing on the development of the liquid phase rate and temperature distribution of the NePCM. The volume fractions of nanoparticles and geometric parameters (wave number of waves) are also compared and studied.

## 2. Problem Description

**Figure 1:**
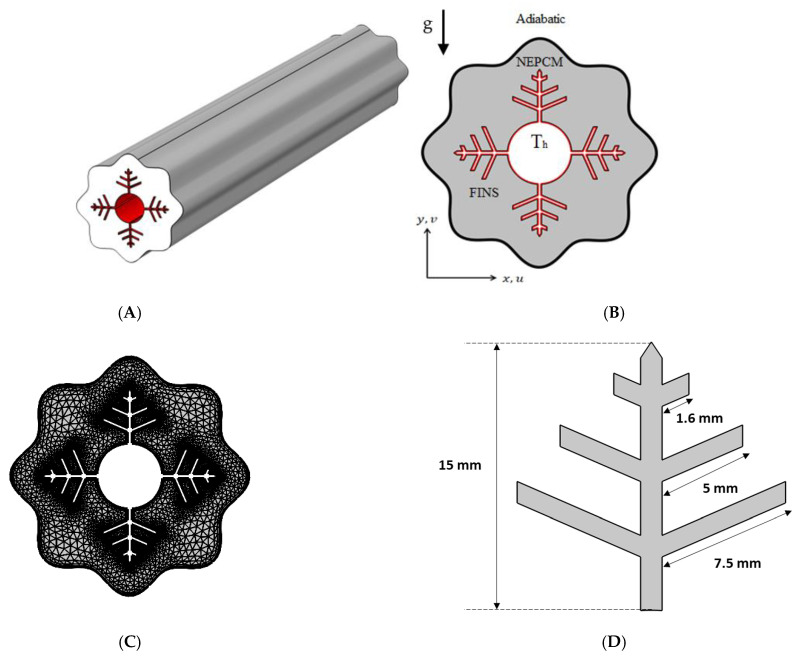
(**A**) Three-dimensional model of the shell and tube TEs with embedded fins; (**B**) A two-dimensional illustration of the studied model with boundary conditions; (**C**) A mesh sample; (**D**) fins dimensions.

Fins, NePCM, and wavy walls are used in this research to expedite the charging process of a shell-and-tube LHTES unit. The analyzed cross-section with boundary conditions is shown in Figure 1, together with a 3D schematic of the shell-and-tube LHTES unit, which is filled with a nano-enhanced PCM composite (Cu/paraffin wax) and fitted with fins. The wavy shell wall is investigated in three examples (N = 4, 6 and 8). The wavy formed shell, and inner tube are 60 mm and 18 mm diameters, respectively. Branching fins are added to enhance the heat propagation into the NePCM, and their dimensions are illustrated in Figure 1D.

According to Xuan et al. [50], nanoparticles with a diameter of less than 100 nm generate a homogenous flow with the base fluid. Therefore, copper nanoparticles with a diameter of 50 nm and volumetric fractions of 0% (pure PCM), 2%, and 4% are investigated.

**Figure 2:**
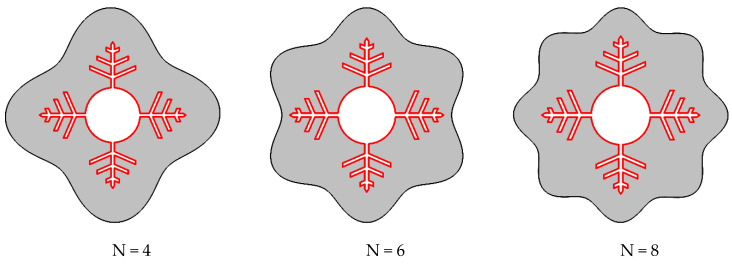
Different cases were considered for N (number of waves in the shell) in this study.

The number of waves in the shell wall was altered while maintaining the container’s volume of PCM constant. The investigated cases are shown in Figure 2.

Paraffin wax is employed as the phase change medium, and copper nanoparticles are utilized to increase the thermal conductivity of the PCM. The thermophysical characteristics of paraffin wax and copper nanoparticles are summarized in Table 1.

### 2.1. Mathematical Model

The enthalpy–porosity methodology is the most frequently used technique for analyzing unstable heat transport problems, such as PCM phase transitions. The enthalpy–porosity technique is utilized for simulating the melting and heat transmission characteristics of embedded fins in NePCM. The enthalpy–porosity approach does not require direct monitoring of the phase contact; instead, it derives the energy equation utilizing enthalpy and temperature throughout the whole calculation domain. Due to the substantial nonlinearity of the phase change process, its issues become more intricate. The general simulation steps include creating the geometry, meshing the geometry, assigning the initial and boundary conditions, solving the momentum equation to obtain the flow field, and solving the energy equation to obtain the thermal field. The following parameters are considered to streamline the computation:

1. The flow of liquid NePCM is regarded to be incompressible and laminar.
2. Overlooking the volumetric impact of viscous dissipation and heat source.
3. The shell wall is assumed to maintain a constant temperature, ignoring the heat transfer resistance of the container wall and the convective heat transfer process inside the tube.
4. There is no heat transfer between the shell and its surroundings.

Based on the above assumptions, it is possible to derive the governing equation for the melting process of NePCM in the wavy finned enclosure. The equations may be expressed as [53,54]:

(1) $$\mathrm{Continuity}\;\mathrm{equation}\;\nabla \cdot \left(\vec{V}\right)=0$$

(2) $$\mathrm{Momentum}\;\mathrm{equation}\;\frac{\partial \left(\rho_{np}\vec{V}\right)}{\partial t}+\nabla \cdot \left(\rho_{np}\vec{V}\right)=-\nabla P+\mu_{np}\nabla^{2}\vec{V}-S_{b}+S_{a}$$

where the subscript *np* refers to the nano-enhanced PCM, $S_{a}$ is the source term for the porosity function proposed by Bernt et al. [55].

(3) $$S_{a}\;\mathrm{define}\;\mathrm{as}\;S_{a}=-A\vec{V}\;\mathrm{with}\;A=\frac{{\left(1-\eta \right)}^{2}}{\left(\pi^{3}+10^{-3}\right)}C$$

(4) $$\mathrm{And}\;\nabla \left(P\right)=-\frac{{\left(1-\eta \right)}^{2}}{\eta^{3}}C\cdot \vec{V}$$

$S_{b}$ is the Boussinesq approximation to the buoyancy force; the value is as follows:

(5) $$S_{b}={\left(\rho_{\;}\beta \right)}_{np}\left(T-T_{m}\right)\vec{g}$$

The vector of fluid velocity is denoted by $\vec{V}$. The two-dimensional model specifies the axial and radial velocity vector components as follows:

(6) $$\;V_{axial\;}=v\;\mathrm{and}\;V_{radial\;}=u\;$$

(7) $$\mathrm{Energy}\;\mathrm{equation}\;\frac{\partial \left(H\right)}{\partial t}+\nabla \cdot \left(\vec{V}H\right)=\nabla \cdot \left(k_{np}\nabla T\right)$$

where *H* signifies a certain enthalpy and is stated in the following manner:

(8) H=h+ΔH

where *h* is a sensible enthalpy denoted by the formula:

(9) $$h=h_{ref}+\int_{T_{ref}}^{T}{\left(\rho c_{p}\right)}_{np}dT$$

the value of C is taken as $C=10^{6}$ [56].

Additionally, η is the equation for the liquid portion of the liquid/solid zone, which assists in defining the zone of calculated cells, where the liquid zone equals η=1 and the solid zone equals η=0, while the mushy zone equals 0<η<1, and can be expressed as follows:

(10) $$\eta \left(T\right)=\left\{\begin{array}{c} 0\;if\;T<T_{s}\;\\ 1\;if\;T>T_{l}\\ \;\frac{T-T_{s}}{T_{l}-T_{s}}\;if\;T_{l}>T\geq T_{s}\; \end{array}\right\}$$

with $T_{l}$ and $T_{s}$ denoting the NePCM’s liquid and solid temperatures, respectively.

It is possible to represent the liquid fraction expression as flows:

(11) $$\eta \left(T\right)=\left\{\begin{array}{c} 0\;if\;T<\left(T_{m}-\Delta T\right)\;\\ 1\;if\;T>\left(T_{m}+\Delta T\right)\\ \;\frac{\left(T-T_{m}+\Delta T\right)}{2\Delta T}\;if\;\left(T_{m}+\Delta T\right)>T\geq \left(T_{m}-\Delta T\right)\; \end{array}\right\}$$

The preceding equations use general notations for the thermophysical characteristics and apply to both pure PCM and NePCM. The thermophysical characteristics of paraffin wax are employed in the situations of pure PCM, and in the cases of NePCM, the parameters are estimated using a mix of paraffin wax and copper nanoparticle properties, as shown in Table 1.

The following equations are used to determine the density and specific heat capacity of the nano-PCM material [57,58]:

(12) $$\rho_{np}=\left(1-\varphi \right)\rho_{p}+\varphi \rho_{n}$$

(13) $${\left(\rho c_{p}\right)}_{np}=\left(1-\varphi \right){\left(\rho c_{p}\right)}_{p}+\varphi {\left(\rho c_{p}\right)}_{n}$$

where *n* and *p* are subscripts for nanoparticles and PCM, respectively.

Here, φ represents the volumetric fraction of nanoparticles added in the PCM.

In a similar manner, latent heat of fusion, the effective thermal conductivity, and the thermal expansion coefficient of NEPCM can be found using the following set of equations.

(14) $${\left(\rho L\right)}_{np}=\left(1-\varphi \right){\left(\rho L\right)}_{p}$$

(15) $$k_{np}=\frac{k_{n}+2k_{p}-2\varphi \left(k_{p}-k_{n}\right)}{k_{n}+2k_{p}+\varphi \left(k_{p}-k_{n}\right)}k_{p}$$

(16) $${\left(\rho \beta \right)}_{np}=\left(1-\varphi \right){\left(\rho \beta \right)}_{p}+\varphi {\left(\rho \beta \right)}_{n}$$

The entropy created as a result of thermal irreversibility (heat transfer) equals

(17) $$S_{ht}=\frac{k_{nf}}{{\bar{T}}^{2}}\left[{\left(\frac{\partial \bar{T}}{\partial x}\right)}^{2}+{\left(\frac{\partial \bar{T}}{\partial y}\right)}^{2}\right]$$

The entropy created as a result of the flow’s irreversibility (presence of a friction factor) is equal to

(18) $$S_{f}=\frac{\mu_{nf}}{\bar{T}}\left\{2\left[{\left(\frac{\partial \bar{u}}{\partial x}\right)}^{2}+{\left(\frac{\partial \bar{v}}{\partial y}\right)}^{2}\right]+{\left(\frac{\partial \bar{u}}{\partial x}+\frac{\partial \bar{v}}{\partial y}\right)}^{2}\right\}$$

The total entropy, which comprises the entropy increase caused by heat transfer and fluid friction, and the Bejan number, which is the ratio of irreversible heat transfer to total entropy, are computed using the following formulae.

(19) $$S_{\mathrm{tot}}=S_{\mathrm{ht}}+S_{f}$$

(20) $$\mathrm{Be}=\frac{S_{\mathrm{ht}}}{S_{\mathrm{tot}}}$$

### 2.2. Validation and Mesh Independence Study

The finite element formulation is used to solve equations using the suitable boundary conditions given above. The weak form of the governing equations is established using the Galerkin method to obtain the finite element formulation. Within each of the motion variables, the computational domain is split into non-overlapping areas, and the interpolation functions are used to approximate them. The velocity components and pressure are discretized using P2–P1 Lagrange finite elements, while the temperature is discretized using Lagrange–quadratic finite elements. When the relative error for each of the variables meets the following convergence conditions, the solution is said to have converged.

|(Γ(n + 1) − Γn)/Γ(n + 1)| ⩽ 10 2212 6

An optimum grid distribution was used for the grid independency research, which yielded correct findings in a short amount of time.

**Figure 3:**
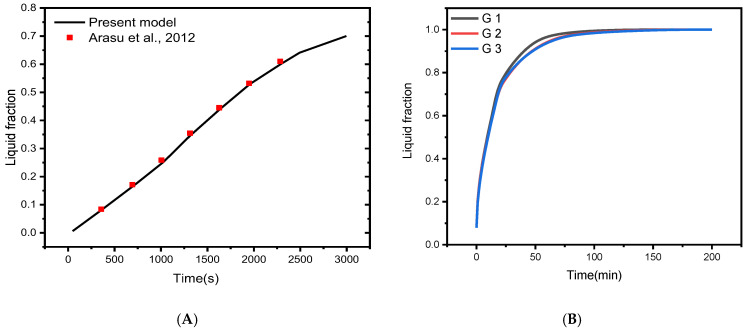
(**A**) Comparison of numerical results with [59]; (**B**) Grid independent study [60].

To verify the implementation of mathematical modeling of melting and the solution methodology outlined above, the result obtained for melting interface propagation in a square enclosure was compared to the simulation solution provided in Arasu et al. [59] in Figure 3A. The present findings are highly consistent with those previously published, implying that the numerical model’s fundamental validity was established.

The mesh independence study is established by examining the average liquid fraction over time for the various grid sizes shown in Table 2. Figure 1C shows an example of the computing grid, where the mesh must be finely tuned throughout the domain to adjust to the moving of the melting interface at each time step. The influence of multiple mesh sizes on the liquid fraction during the melting process is shown in Figure 3B. The mesh G2 is selected to conduct all numerical simulations in this research based on the outcomes of the mesh independence study (illustrated in Figure 3B).

## 3. Results and Discussion

In this research, the objective is to optimize the phase transition mechanism inside a shell and tube TES unit while accelerating the process of thermal energy charging. Accordingly, Cu nanoparticles are introduced to paraffin wax PCM at varying concentrations, and the TES unit employs fins with a snowflake design. The effect of these variables on the needed melting time is examined in terms of temperature distribution, fraction liquid, Bejan number (the ratio of heat transfer contribution to total entropy), average Nusselt number, average temperature, average Bejan number, and average fraction liquid. To obtain more detailed results elaborating the acceleration of the NePCM melting process and heat transfer enhancements, the average Nusselt number (Nu_avg_), average Bejan number (Be_avg_), the average liquid fraction (B), and the average temperature in the TES unit are interpreted for various nanoparticle concentrations and wavenumbers.

### 3.1. Time Needed for Melting NePCM

**Figure 4:**
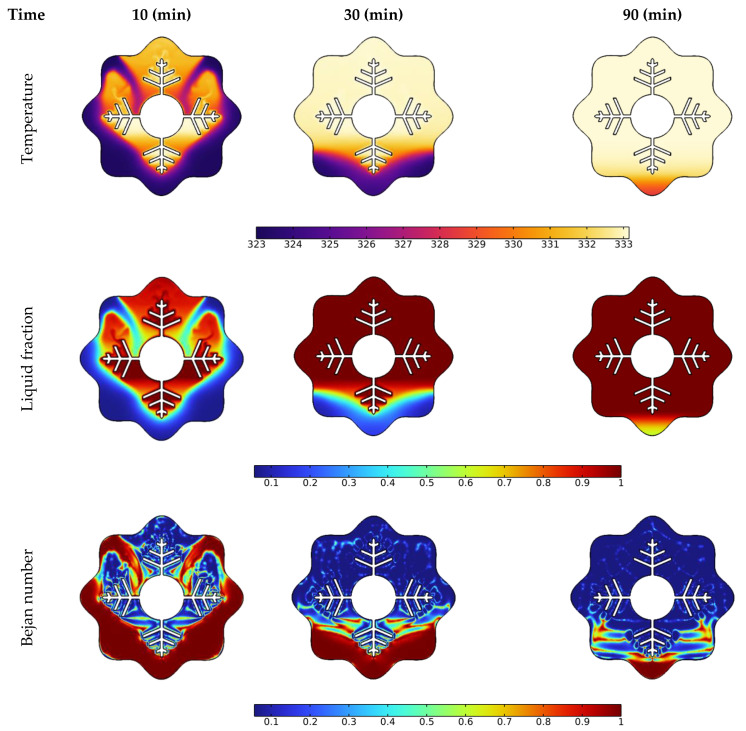
Effect of time on temperature, liquid fraction, and Bejan number during the PCM melting process.

Temperature, liquid fraction, and Bejan number contours between the wavy shell and finned tube at different stages throughout the melting process are depicted in Figure 4. The blue and red colors in the second row represent the solid and liquid PCMs, respectively. The higher temperature areas are found near the tube and fin surfaces at the early stage of melting (during the first 10 min), and a layer of melted PCM forms around them. Over the following 30 min, the temperature values in NEPCM around the fins and the upper half of the annulus rise, and heat penetrates rapidly into the solid layers of the PCM from the fin’s wall. The thickness of the layer of melted PCM increases rapidly as time passes, however, a solid zone is always present at the bottom. Due to the existence of natural convection, the melting pace at the top portion of the examined annulus is much greater than that at the bottom section. With sustained heating, the melting continues at the bottom of the unit and, after 90 min, approximately the whole volume of NEPCM was melted, except for a little portion in the bottom corner.

The Bejan number (the third low in Figure 4) demonstrates a drop in heat transfer contribution to total entropy as time passes. The main reason for this drop is that the PCM has reached a stable temperature in many locations within the annulus, as seen in the third column at 90 min. Since this is a temperature difference-dependent heat transfer phenomenon. As a result, entropy generation contributed by heat transmission is reduced when the temperature difference decreases.

### 3.2. Nanoparticle’s Concentration Effect on the Acceleration of the Melting Process

The melting increase caused by the addition of nanoparticles to the PCM (NEPCM) is investigated in this subsection using the configuration of N = 6. Cu nanoparticles are spread in paraffin wax, functioning as the PCM in volumetric quantities of 0% (pure PCM), 2%, and 4%. The PCM’s enhanced thermophysical properties are determined using the model equations given in Section 2 and the properties provided in Table 1.

**Figure 5:**
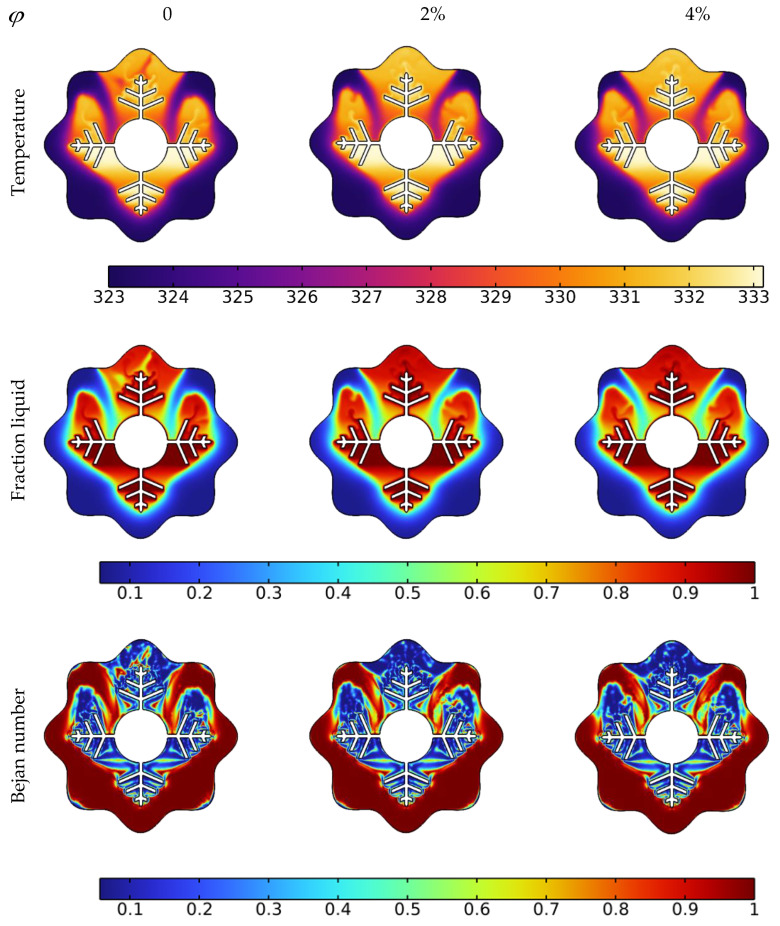
Impact of Cu nanoparticle concentration on temperature, liquid fraction, and Bejan number during the PCM melting process after 10 min.

**Figure 6:**
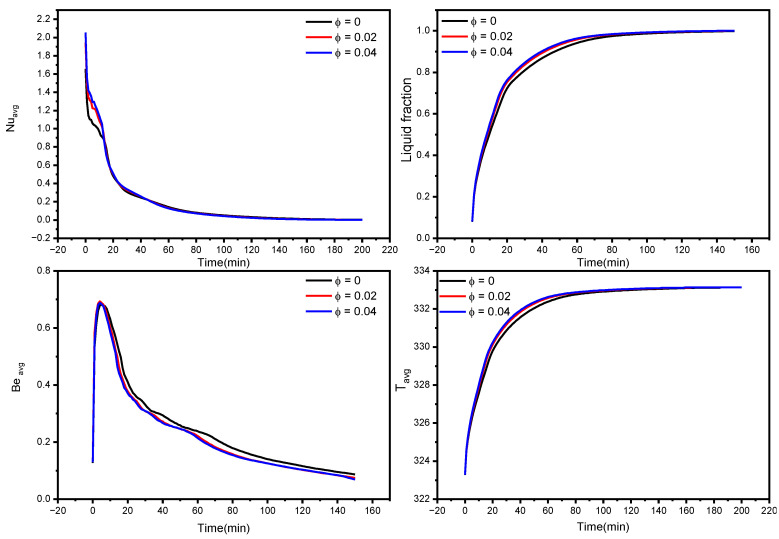
Influence of Copper nanoparticles concentration on average Nusselt number, the average liquid fraction (B), Bejan number, and average temperature during the NePCM melting process.

The liquid fraction, temperature, and Bejan number distribution after 10 min with various nanoparticle concentrations in the PCM for N = 6 are shown in Figure 5. The average Nusselt number (Nu_avg_), average Bejan number (Be_avg_), the average liquid fraction (B), and the average temperature in the TES unit are shown in Figure 6. As a reference, the findings of pure PCM are also presented.

Copper nanoparticles are very conductive, and their inclusion in the PCM may remarkably improve its thermal conductivity. As a consequence of the higher thermal conductivity, the conduction heat transfer rates during the melting process increase, culminating in faster melting. It is particularly important during the first melting phase, as well as later in the melting process when only the solid pool lingers at the bottom. The gradual increment in thermal conductivity with a rise in nanoparticle concentration, hence improving heat transmission, generates a rapid temperature rise. Moreover, the entropy production associated with heat transfer is enhanced, resulting in higher values of the Bejan number, as seen in the third row of Figure 5.

Larger concentrations of NEPCM result in faster melting rates. Furthermore, as seen in Figure 6, the NEPCM samples melt quicker during the melting process, owing to their increased heat conductivity. The whole volume of the NEPCM melts in 153 min and 148 min for samples with 2% and 4% nanoparticle concentrations, respectively, compared to 173 min for the pure PCM sample. With increasing concentrations, Nu_avg_ and Be_avg_ values decreased, which could be attributed to the fact that introducing nanoparticles to the PCM bolstered its thermal properties, increasing heat transfer and melting rate, implying that the temperature in NEPCM samples became uniform faster as time passed (leading to a smaller temperature gradient, as seen in Figure 6 bottom right). As a result, the heat transmission intensity reduces, and Nu_avg_ and Be_avg_ fall quicker in NEPCM than in pure PCM.

### 3.3. Influence of Waves Number on the Melting Process

**Figure 7:**
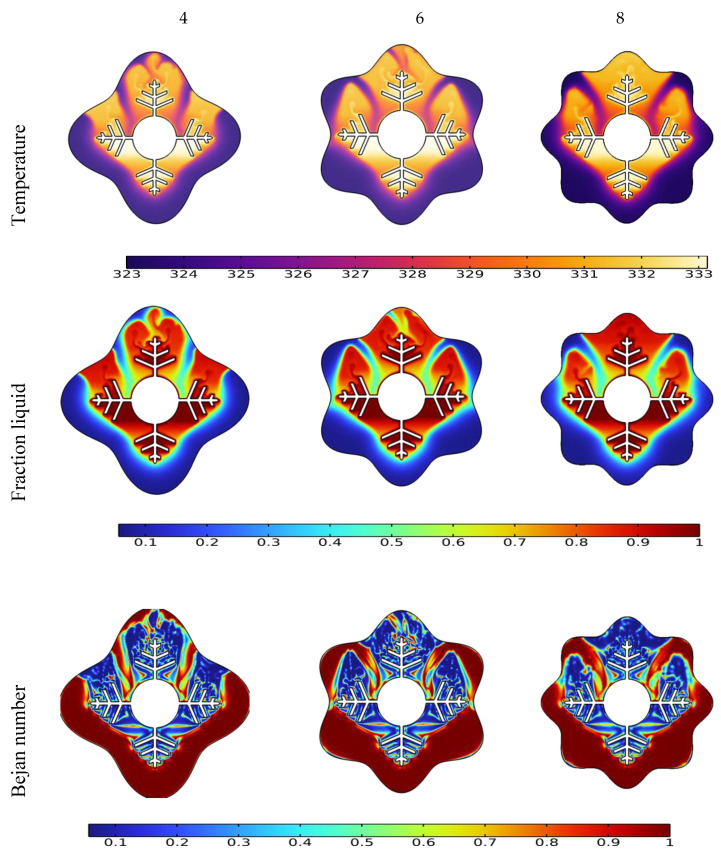
Effect of wave number N on temperature, liquid fraction, and Bejan number during the PCM melting process after 10 min.

**Figure 8:**
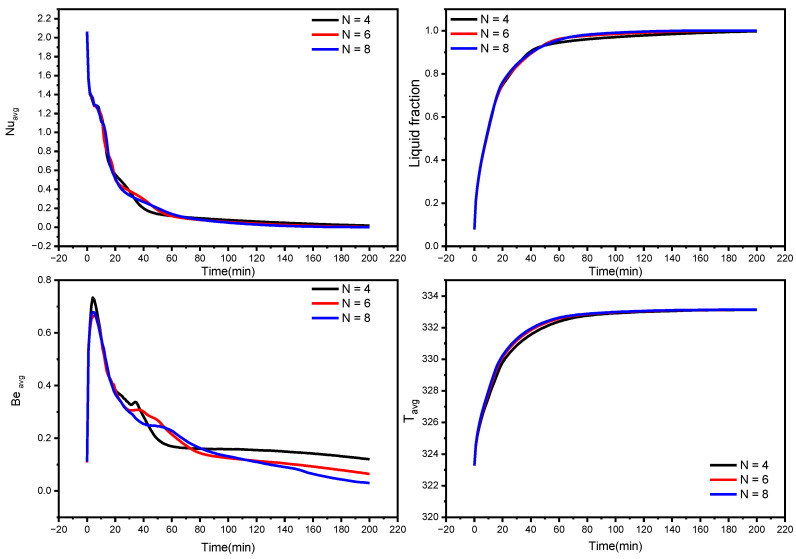
Influence of wave number on average Nusselt number, the average liquid fraction (B), Bejan number, and average temperature during the NePCM melting process.

The liquid fraction, temperature, and Bejan number distribution after 10 min for various wavenumbers (N = 4, 6, and 8) are shown in Figure 7. The average Nusselt number (Nu_avg_), average Bejan number (Be_avg_), the average liquid fraction (B), and the average temperature in the TES unit are shown in Figure 8.

The form of the PCM containers is critical in accelerating the melting process for the TES unit [51]. This section discusses the effect of different wave numbers (N = 4, 6 and 8) at 10 min time mark. Figure 7 illustrates that the N = 8 configuration shows a higher temperature difference and liquid fraction at the top region of the TES unit since it shrinks the distance between the fin and external surface. Additionally, the Bejan number values confirm the minimized heat transfer losses in the regions that have relatively uniform temperature distribution.

**Figure 9:**
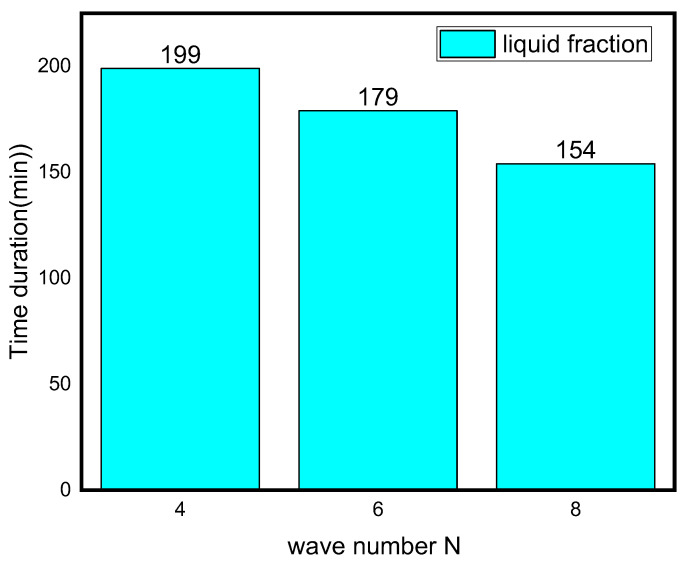
Time duration for different wave numbers.

The effect of wave number on heat transfer is shown in Figure 8 and Figure 9 in terms of Nu_avg_, Be_avg_, the average liquid fraction (B), and T_avg_ as a function of time. Figure 8 illuminates that heat transfer for N = 6 and N = 8 is superior to N = 4 after the first 45 min, which is a critical period in melting propagation, especially for the bottom region. N = 8 design minimizes the distance between the fins and the external surface, which minimizes the thermal resistance of NEPCM. The full body of the PCM takes 227, 180, and 155 min to melt for N = 4, N = 6, and N = 8, respectively. This indicates that using the N = 8 design rather than the N = 4 design reduces the melting time by 31%. The Be_avg_ is high for the first 25 min in all circumstances owing to elevated heat transfer rates, then it subsequently drops as the temperature gradient progressively diminishes, which is indicated by the T_avg_ plot in Figure 8.

## 4. Conclusions

The numerical simulation of the melting of PCM inside an annulus between a wavy shell and finned tube of the TES unit is provided in this article. The impacts of the wavy shell and nanoparticle addition on the melting rate and heat transmission characteristics were examined. The outcomes demonstrate that:

- Owing to the presence of natural convection, the melting rate seen in the upper section of the analyzed annulus is significantly higher than that observed at its bottom portion;
- Depending on the volume fraction of nano-additive used, the melting time could be reduced from 3% to 14% with 2% and 4% nanoparticle concentrations, respectively;
- The phase transition process may be greatly accelerated by increasing the wave number N. When the wave number was increased from four to eight, the overall melting time decreased by 31%.

## Figures and Tables

**Table 1:** Thermophysical proprieties of (PCM), inner-tube wall, and Copper nanoparticles. Data from [51,52].

Properties of the Materials	PCM		Inner-Tube Wall	Nanoparticle
	(paraffin wax)		Copper	Copper
	Solid	Liquid		
**Thermal conductivity, *k*, (W/mK)**	0.39	0.157	401	401
**Density, *ρ*, (kg/m^3^)**	775	833.6	8900	8954
**Kinematic viscosity (m^2^/s)**	8.31 × 10^−5^			
**Thermal expansion coefficient, *β*, (1/K)**	-	7.14 × 10^−3^		1.67 × 10^−5^
**Specific heat, *c_p_*, (kJ/kgK)**	2.44	2.384	0.385	0.385
**The melting point (°C)**	54.32			
**Latent heat of fusion (kJ/kg)**	184.48			
**The reference temperature (°C)**	50			

**Table 2:** Numerical test results for grid independence study.

Mesh	G1	G2	G3
**Number of elements**	25,096	66,982	100,384

## Data Availability

Not applicable.
